# Double-Lumen Intubation Facilitating a Single-Anesthesia Workflow in Robot-Assisted Navigational Bronchoscopy and Subsequent Lung Resection: A Single-Center, Retrospective Study

**DOI:** 10.3390/jcm15031025

**Published:** 2026-01-27

**Authors:** Hruy Menghesha, Jan Arensmeyer, Philipp Feodorovici, Mark Coburn, Dirk Skowasch, Daniel Kütting, Joachim Schmidt, Donatas Zalepugas

**Affiliations:** 1 Department of Thoracic Surgery, University Hospital Bonn, Venusberg-Campus 1, 53127 Bonn, Germany; 2 Department of Thoracic Surgery, Helios Clinic Bonn/Rhein-Sieg, 53123 Bonn, Germany; 3 Bonn Surgical Technology Center (BOSTER), University Hospital Bonn, Joseph-Schumpeter-Allee 1, 53227 Bonn, Germany; 4 Department of Anaesthesiology and Intensive Care Medicine, University Hospital Bonn, 53127 Bonn, Germany; 5 Department of Internal Medicine II—Pneumology/Cardiology, University Hospital Bonn, 53127 Bonn, Germany; 6 Department of Diagnostic and Interventional Radiology, University Hospital Bonn, Venusberg-Campus 1, 53127 Bonn, Germany

**Keywords:** lung cancer screening, robotic navigational, bronchoscopy, lung nodule, double lumen tube

## Abstract

**Background:** Robotic-assisted navigational bronchoscopy (RNB) using the ION system (Intuitive Surgical, Sunnyvale, CA, USA) combined with cone-beam computed tomography (CBCT) (Cios Spin, Siemens Healthineers, Erlangen, Germany) and tool-in-lesion verification enables precise diagnosis of peripheral pulmonary nodules. Integrating RNB with intraoperative frozen section analysis may allow same-day resection, avoiding delays between diagnosis and treatment. Standard airway management with a single-lumen tube (SLT) limits immediate transition to lung resection, whereas initial double-lumen tube (DLT) placement could streamline workflow and improve safety. This study evaluated the diagnostic performance, procedural efficiency, and feasibility of an integrated ION-guided RNB workflow using either SLT or DLT. **Methods:** In this single-center retrospective study, 36 consecutive patients undergoing ION-guided RNB for pulmonary nodules between August 2024 and June 2025 were analyzed. Airway management (SLT vs. DLT) was selected based on surgical planning. Lesions were targeted using CBCT or C-arm fluoroscopy, and biopsies were performed via forceps or cryoprobes. Frozen section results guided immediate surgical resection when malignancy was confirmed. **Results:** Thirty-six patients (mean age 64.9 ± 7.9 years; female/male ratio 16/20) with 42 nodules (mean diameter 1.22 ± 0.76 cm) were included; 76.2% were peripheral. Mean RNB time was 58.3 ± 21.3 min. Overall diagnostic yield was 73.0%, significantly higher with DLT versus SLT (84.2% vs. 50.0%, *p* = 0.035), with more biopsies per patient (7.9 ± 2.2 vs. 3.2 ± 3.1, *p* = 0.035). No major complications occurred. **Conclusions:** ION-guided RNB with CBCT and intraoperative frozen section enables accurate, single-session diagnosis and treatment of pulmonary nodules. Upfront DLT placement facilitates procedural efficiency within a streamlined “one-stop-shop” workflow without compromising diagnostic yield.

## 1. Introduction

Peripheral pulmonary nodules (PPNs) present a diagnostic challenge due to the critical need for early detection—particularly in malignant cases—balanced against the risks of invasive procedures. The detection of subclinical nodules has increased significantly as a result of the broad use of low-dose computed tomography (CT) screening. Nevertheless, suboptimal diagnostic yield or high complication rates often limit standard diagnostic procedures, such as transthoracic needle biopsy (TTNB) or conventional bronchoscopy [1,2,3]. The impending implementation of population-wide lung cancer screening programs further underscores the demand for credible, minimally invasive diagnostic procedures. Navigation-assisted bronchoscopy has also improved access to peripheral areas of the lung; however, mechanical restrictions can impair sampling success [4,5].

Recently, robot-assisted navigational bronchoscopy (RNB), and specifically with the ION system (Intuitive Surgical, Sunnyvale, CA, USA), appears to have the potential to overcome these drawbacks due to its ability for precision-controlled, catheter navigation with increased reach, and to allow real-time, three-dimensional confirmation of tool-in-lesion placement with cone-beam CT (CBCT) [6,7,8,9]. When combined with intraoperative frozen section analysis for histopathological evaluation, malignancy can subsequently be confirmed, and surgical resection of lung cancer can proceed without interruption in the same anesthetic episode, thus avoiding a second intervention in the same patient [10,11,12].

One of the challenges for diagnostic RNB to be incorporated with immediate surgical resection is that conventional airway management is limited. The ION system is typically utilized in combination with a single-lumen endotracheal tube (SLT), which is reasonable for diagnostic procedures [13]. However, if during the procedure, the frozen section indicates localized non-small cell lung cancer, and immediate anatomical resection is recommended, there are logistical and clinical obstacles for the workflow in the operating room. Anatomical lung resections require single-lung ventilation (SLV), which cannot be accomplished sufficiently with an SLT without a bronchial blocker [14,15]. In such scenarios, reintubation with a double-lumen tube (DLT) becomes necessary—causing procedural delays, interrupting workflow, and increasing the complexity and duration of the operation. Moreover, the reintubation process creates additional risks from airway manipulation while the patient is under general anesthesia, and it increases the difficulty of intubation, particularly for patients who may have oxygenation challenges due to chronic pulmonary or cardiovascular comorbidities [16,17]. Such disruptions directly contradict the fundamental aim of the “one-stop-shop” model: to consolidate diagnosis and treatment within a single anesthetic episode while minimizing time to therapy.

In this context, we believe that the use of a double-lumen endotracheal tube from the beginning of the combined diagnostic–therapeutic procedure may be a pragmatic and useful approach. Ultimately, the use of a double-lumen tube allows for an easy transition from diagnostic bronchoscopy to surgical resection and would avoid the need for reintubation if lung isolation use was required for surgical resection. The change we are proposing may increase intraoperative efficiency, decrease the duration of anesthesia and risk for the patient, and, overall, improve the feasibility of the “one-stop-shop” approach in treating peripheral pulmonary nodules.

Timely initiation of definitive therapy is still an important determinant of outcome in lung cancer management. While no universal maximum time period between diagnosis and treatment has been established, national and international guidelines (German S3, ESMO, ESTS, NCCN, and ASCO) consistently highlight the importance of minimizing delays through rapid diagnostics and multidisciplinary planning. [18,19,20].

This focus may reflect the increasing awareness that delays in treatment coincide with worse oncologic outcomes, generally because of the increased risk of either disease progression or metastasis [21,22]. Consequently, healthcare systems are under mounting pressure to optimize diagnostic and treatment pathways for thoracic oncological patients.

This streamlined process incorporates multiple diagnostic or therapeutic steps into a single session and could also potentially shrink the time from detection to treatment from weeks to days. We believe that minimizing the time interval from diagnosis to definitive therapy could improve clinical outcomes by minimizing the chance of interim tumor growth or upstaging.

While emerging studies have begun to evaluate the diagnostic performance of RNB and explore intraoperative workflows that combine diagnostic and therapeutic interventions [10,23], data remain scarce on the implementation and practical benefits of an integrated “one-stop-shop” strategy. The current study aims to address this gap in the literature by examining the diagnostic accuracy, procedural efficiency, and intraoperative decision-making of an integrated workflow based on ION-guided RNB, CBCT verification, frozen section analysis, and same-day resection as appropriate. Further, we considered the operational and economic ramifications of changing the traditional anesthetic plan—more specifically, double-lumen intubation—to optimize transitions between procedures.

## 2. Materials and Methods

### 2.1. Study Design and Patient Selection

This retrospective cohort study included all 36 consecutive patients undergoing ION™-guided (Intuitive Surgical, Sunnyvale, CA, USA) RNB at our tertiary thoracic surgical center between August 2024 and June 2025. All adult patients with detected lung nodules referred for diagnostic bronchoscopy were considered.

The study was approved by the ethics board (2025-417-BO). All patient data included in this analysis were fully available at the time of data collection. The data were obtained exclusively from the institution’s electronic medical records. No prospective data acquisition was performed. Due to the retrospective nature of the study and the complete anonymization of all datasets, individual patient consent was not required in accordance with applicable data protection regulations and ethical guidelines.

The study was conducted in accordance with the ethical principles outlined in the Declaration of Helsinki.

### 2.2. Preoperative Preparation

All patients sent to our center with pulmonary nodules noted on chest CT were evaluated based on the anatomy and estimated risk of malignancy. Each case was reviewed and discussed in a multidisciplinary tumor board. When an indication for tissue diagnosis was made in the interdisciplinary meeting, the patient had navigational bronchoscopy scheduled using the ION™ system (Intuitive Surgical, Sunnyvale, CA, USA), with the decision regarding subsequent surgical resection made based on the histopathological results. Each case underwent a complete physical exam, medical history review, and guideline-conforming functional workup to determine potential for upcoming lung resection. This consisted of body plethysmography with lung function measurement for carbon monoxide diffusion capacity (DLCO), 12-lead surface electrocardiogram (ECG), and laboratory work assessing vital parameters, including white blood cells, hemoglobin, renal retention, and infection markers. In cases where pathological findings were identified, further diagnostic evaluations—such as transthoracic echocardiography or cardiopulmonary exercise testing—were conducted as clinically indicated.

### 2.3. Procedural Workflow

**Figure 1:**
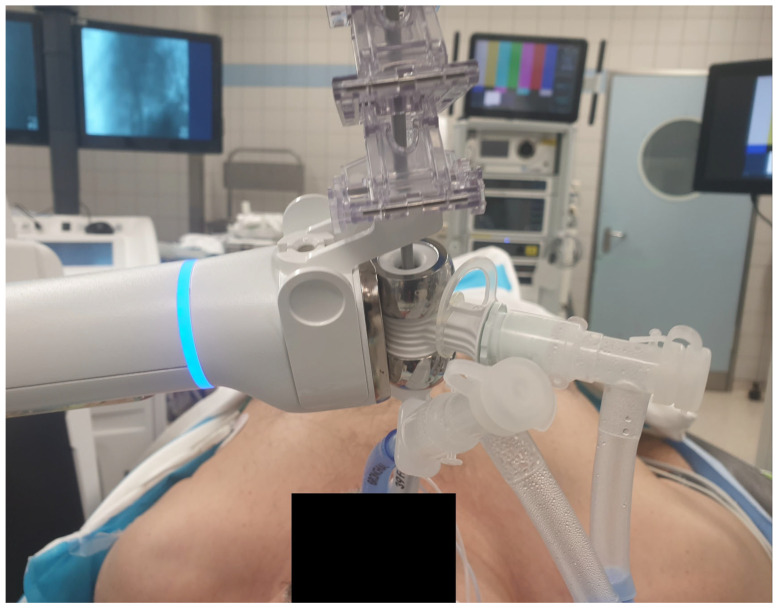
Illustration of a patient undergoing an ION biopsy procedure. The ION system (Ion Endoluminal System, Intuitive Surgical) is connected to the tracheal limb of the double-lumen tube.

All patients were intubated and managed intraoperatively by an attending anesthesiologist, with the choice of endotracheal tube for intubation—double-lumen (DLT) (Figure 1) or single-lumen (SLT)—based on the surgeon’s discretion pre-operatively, based on the likelihood of lung resection. As these were the first consecutive patients treated by this surgeon at this center using the ION™ system, the procedures were initially done without the routine use of DLT. All procedures were performed by the same attending surgeon. Target navigation was achieved with the ION™ system, which included virtual maps based on CT images. The first cases did not have cone-beam computed tomography (CBCT) (Cios Spin, Siemens Healthineers, Erlangen, Germany) available at the start of our ION program, and thus, the targets were localized using C-arm fluoroscopy, with subsequent cases using CBCT. The biopsy tools were selected based on the nodule characteristics, using forceps, cryoprobes, or both. Intraoperative frozen sections were routinely performed, and if there was malignancy or high suspicion of malignancy, definitive lung resection was performed.

### 2.4. Histopathological Workup

All tissue samples obtained intraoperatively via the ION system were first evaluated using frozen section analysis, followed by formalin fixation and paraffin embedding (FFPE) for further examination. When patients proceeded to definitive surgical resection after biopsy confirmation, the resected specimens were similarly processed as FFPE samples. The conclusive histopathological diagnosis was made according to the latest WHO classification guidelines.

### 2.5. Subgroup Analysis

To evaluate the differences in intraoperative outcomes based on the method of intubation, patients were divided into two groups: DLT (double-lumen tube) and SLT (single-lumen tube). The analysis focused on procedure duration, number of biopsies obtained, tumor size, diagnostic yield, complications, and the type of intraoperative imaging used. To eliminate the evident bias introduced by the use of the C-arm, this patient subgroup was excluded, and the analysis was repeated for the remaining patients based on their initial method of intubation.

### 2.6. Statistical Analysis

Statistical analysis was conducted using IBM SPSS Statistics for iOS, Version 29.0 (IBM Corp., Armonk, NY, USA). Descriptive statistics were used to summarize patient demographics, lesion characteristics, and procedural variables. Diagnostic yields were compared between subgroups using chi-squared and Fisher’s exact tests. A *p*-value < 0.05 was considered statistically significant.

## 3. Results

### 3.1. Patient and Lesion Characteristics

A total of 36 patients and 42 lung nodules were included in this study. Of all the patients, 16 (44.4%) were female. The mean age of the cohort was 64.94 ± 7.94 years. Most lesions (32; 76.2%) were located in the peripheral third of the lung, while 6 (14.3%) were situated in the middle third and 4 (9.5%) in the central third. With regard to anatomical distribution, 11 (26.2%) of lesions were located in the left lower lobe (LLL), 12 (28.6%) in the left upper lobe (LUL), 11 (26.2%) in the right upper lobe (RUL), and 4 (9.5%) in either the middle lobe (ML) or the right lower lobe (RLL). The mean nodule diameter was 1.22 ± 0.76 cm. Radiologically, 33 (78.6%) lesions were classified as solid, while 8 (19.0%) presented as ground-glass opacities (GGO) and 1 (2.4%) was sub-solid (Table 1).

### 3.2. Procedural Metrics

The mean total procedure time for robotic navigational bronchoscopy (RNB) was 58.28 ± 21.32 min. Cone-beam computed tomography (CBCT) was used for intraoperative guidance in 33 (78.6%) of cases, while C-arm fluoroscopy was used in the remaining 9 (21.4%). The average number of biopsies obtained per patient was 5.39 ± 3.59. Biopsy techniques included forceps in 40.0% of cases, cryoprobe in 48.0%, and a combination of both in 12.0% (Table 1).

### 3.3. Intraoperative Management and Complications

Among all patients, 16 (44.4%) were intubated with a single-lumen tube (SLT), while 20 (55.6%) received upfront double-lumen tube (DLT) placement. A total of 88.9% of patients proceeded to surgical resection following diagnostic confirmation. Of these, 58.3% underwent robotic-assisted thoracoscopic surgery (RATS) and 30.6% underwent video-assisted thoracoscopic surgery (VATS). One patient experienced endobronchial bleeding, which was managed conservatively. No major intraoperative complications were observed (Table 1).

### 3.4. “Tool-in-Lesion” and Diagnostic Yield

“Tool-in-lesion” could be achieved in all patients where CBCT was used. The overall diagnostic yield across all patients was 73.0% (Table 1).

### 3.5. Comparison Between SLT and DLT

After stratification based on the airway management technique employed, 16 patients underwent RNB with SLT and 20 with DLT. There was no statistically significant difference in mean procedure time between the SLT and DLT groups (63.06 ± 18.64 min vs. 54.45 ± 23.02 min, *p* = 0.234). However, the number of biopsies obtained per patient was significantly higher in the DLT group (7.9 ± 2.2) compared to the SLT group (3.2 ± 3.1, *p* = 0.035). Lesion size was comparable between the groups (1.59 ± 0.84 cm in the SLT group vs. 1.12 ± 0.66 cm in the DLT group, *p* = 0.070). Notably, diagnostic yield was significantly higher in the DLT group (84.2%) than in the SLT group (50.0%) (*p* = 0.035). No serious adverse events or adverse events were observed. A minor endobronchial hemorrhage occurred in the SLT group and was effectively controlled by local administration of epinephrine (Table 1). Use of C-arm fluoroscopy was significantly more frequent in the SLT group (56.3%) compared to none in the DLT group (*p* = <0.001) (Table 2).

### 3.6. SLT vs. DLT (Excluding C-Arm Cases)

After excluding patients who underwent localization using a C-arm, 27 patients remained, comprising 7 in the SLT group and 20 in the DLT group. Mean procedure times remained comparable between the two groups (63.28 ± 21.45 min for SLT vs. 54.45 ± 23.03 min for DLT, *p* = 0.383). While the average number of biopsies per patient was higher in the DLT group (7.9 ± 2.2) than in the SLT group (5.1 ± 3.1), this difference did not reach statistical significance (*p* = 0.060). Lesion size remained similar between groups (1.23 ± 0.39 cm for SLT vs. 1.12 ± 0.66 cm for DLT, *p* = 0.675). Diagnostic yield remained higher in the DLT group (84.2%) compared to the SLT group (66.7%), though this difference was without statistical significance (*p* = 0.562).

## 4. Discussion

This study illustrates the feasibility and clinical utility of an integrated “one-stop-shop” workflow in the management of peripheral pulmonary nodules (PPNs), incorporating robotic navigational bronchoscopy (RNB), intraoperative CBCT, intraoperative frozen section pathology, and, in suitable cases, immediate surgical resection. Notably, we found that upfront use of a double-lumen tube (DLT) intubation not only allowed for an easy intraoperative transition to lung resection but was not linked to a lower diagnostic yield compared to single-lumen tube (SLT) intubation. Overall, our cohort achieved a diagnostic yield of 73% with RNB, which is consistent with the previously reported range of 57–88%, depending on adjunct imaging modalities, lesion type, and the endoscopist’s experience level [7,8,24,25]. In our DLT group, we achieved a diagnostic yield of 84.2%, which is among the highest reported to date, including rates reported before the use of real-time CBCT, suggesting that the combination of advanced imaging support and procedural integration contributes to optimized tissue acquisition, rather than the airway strategy alone.

Importantly, the observed higher diagnostic yield in the DLT group must be interpreted with caution. The DLT cohort was more frequently supported by intraoperative CBCT, whereas the SLT group predominantly relied on conventional C-arm fluoroscopy during the early study phase (Table 2).

To address the potential confounding effect of imaging modality, we performed a subgroup analysis excluding SLT cases performed with C-arm fluoroscopy only (Table 3). When comparing SLT and DLT procedures supported by CBCT, diagnostic yield remained numerically higher in the DLT group (84.2% vs. 66.7%), but this difference was no longer statistically significant. This finding suggests that advanced imaging, rather than the airway interface itself, is the dominant determinant of diagnostic success, while DLT does not compromise yield.

Our results reinforce the pivotal role of advanced imaging support. CBCT, which is used in 75% of cases, continues to show a benefit to tool-in-lesion confirmation and diagnostic yield [26,27]. As expected, the use of C-arm fluoroscopy, which was limited to the initial time period in which we only used the SLT, was associated with markedly lower diagnostic performance. In addition, these earlier SLT procedures were performed during the initial learning curve of robotic bronchoscopy at our institution, which may have further contributed to the lower diagnostic yield observed in this group. After excluding SLT procedures performed with C-arm fluoroscopy, diagnostic yield remained numerically higher in the DLT group, although the difference was not statistically significant. These findings underscore the multifactorial nature of diagnostic success in peripheral bronchoscopy, with airway management, imaging guidance, and procedural expertise interacting synergistically, but also show that intraoperative economy does not compromise diagnostic performance.

A significant advantage of our approach is the operational efficiency. The “one-stop-shop” model reduces time to diagnosis and allows immediate resection upon intraoperative confirmation of malignancy. This is of high clinical relevance, given the growing evidence linking delays in treatment to worse oncologic outcomes, particularly in early-stage non-small cell lung cancer (NSCLC) [28]. By enabling diagnosis and curative surgery in a single session, this workflow supports timely care delivery and may improve survival, patient satisfaction, resource utilization, and cost-efficiency [23].

DLT was not associated with increased procedural time or complication rates. Moreover, no periprocedural complications were documented in the DLT group, whereas minor bleeding occurred in the SLT group. While the small sample size precludes firm statistical conclusions, these findings suggest that DLT is not inferior to SLT in terms of safety. Prior studies have highlighted the risks of reintubation under general anesthesia, particularly in patients with compromised pulmonary reserve [29].

Consequently, this study was not designed to demonstrate the superiority of one airway strategy over another in terms of diagnostic yield. Instead, our findings indicate that DLT-based workflows do not compromise diagnostic performance when compared with SLT-based approaches, while offering substantial logistical advantages.

## 5. Conclusions

This study demonstrates the feasibility and safety of incorporating double-lumen tube intubation into an integrated robotic bronchoscopy workflow for peripheral pulmonary nodules. While diagnostic yield was high in the DLT group, this finding should be interpreted in the context of concomitant advanced imaging support. Importantly, DLT was not associated with inferior diagnostic performance, increased procedural time, or higher complication rates. Its use enables seamless transition to same-session surgical resection, supporting a “one-stop-shop” strategy that may improve procedural efficiency and patient care.

## 6. Limitations

Several limitations warrant consideration. First, this was a retrospective, single-center analysis with a relatively small sample size, limiting generalizability and statistical power, particularly for subgroup comparisons. Second, airway management was not randomized but determined by preoperative surgical planning and anticipated need for same-session resection, introducing significant selection bias. Lesions with a higher pre-test probability of malignancy were more likely to be attributed to the DLT group, which may have skewed both diagnostic yield and resection rates in favor of this cohort. In addition, the gradual transition from C-arm fluoroscopy to CBCT during the study period complicates an isolated evaluation of airway strategy effects. While a subgroup analysis excluding C-arm–guided SLT procedures was performed, differences in imaging modality likely represent a major driver of diagnostic performance and limit attribution of yield differences to tube type alone. Finally, diagnostic success is influenced by multiple interacting procedural factors, and the observed higher yield in the DLT group should therefore not be interpreted as a direct effect of the airway interface.

## 7. Future Directions

Prospective multicenter studies are needed to validate these findings and to define clear selection criteria for airway management in diagnostic–therapeutic bronchoscopy workflows. Cost-effectiveness analyses studies may also help quantify institutional benefits and support broader implementation of DLT-based “one-stop-shop” protocols. Additionally, further exploration of alternative airway tools—such as bronchial blockers—may expand the range of feasible techniques.

## Figures and Tables

**Table 1:** Baseline characteristics.

	Total (*n* = 36 Patients/*n* = 42 Nodules)
**Age (patients)**	64.94 ± 7.94
**Female (patients)**	16 (44.4%)
**Topographical data (nodules)**	
Peripheral third	32 (76.2%)
Middle third	6 (14.3%)
Central third	4 (9.5%)
**Lesion Lobe (nodules)**	
RUL	11 (26.2%)
ML	4 (9.5%)
RLL	4 (9.5%)
LUL	12 (28.6%)
LLL	11 (26.2%)
**Morphological data (nodules)**	
Lesion size (cm)	1.22 ± 0.76
**Lesion Density (nodules)**	
Solid	33 (78.6%)
Sub-solid	1 (2.4%)
GGO	8 (19.0%)
**Imaging tool (nodules)**	
C-arm	9 (21.4%)
CBCT	33 (78.6%)
**Primary airway management (patients)**	
SLT	16 (44.4%)
DLT	20 (55.6%)
**Biopsy tool (nodules)**	
Forceps	40.0%
Cryoprobe	48.0%
Cryoprobe + Forceps	12.0%
**Number of biopsies per patient**	5.39 ± 3.59
**Procedure time (min.)**	58.28 ± 21.32
**Surgical technique (%)**	
**RATS**	58.3
**VATS**	30.6
**None**	11.1
**Diagnostic yield (%)**	73.0
**(Serious) Adverse Event**	**0**%
**Minor bleeding**	**1 (2.4%)**

Abbreviations: CBCT, cone-beam computed tomography; DLT, double-lumen tube; RATS, robot-assisted thoracoscopic surgery; SLT, single-lumen tube; VATS, video-assisted thoracoscopic surgery.

**Table 2:** SLT vs. DLT.

	SLT (*n* = 16)	DLT (*n* = 20)	*p*-Value
**Procedure Time (min.)**	63.06 ± 18.64	54.45 ± 23.02	0.234
**Number of biopsies per patient**	3.16 ± 3.07	7.88 ± 2.23	0.035
**Lesion size (cm)**	1.59 ± 0.84	1.12 ± 0.66	0.070
**Diagnostic yield (%)**	50.0	84.2	**0.035**
**C-arm**	**9 (56.3%)**	**0.0**	**<0.001**

**Table 3:** SLT vs. DLT without C-arm.

	SLT (*n* = 7)	DLT (*n* = 20)	*p*-Value
**Procedure Time (min.)**	63.28 ± 21.45	54.45 ± 23.03	0.383
**Number of biopsies per patient**	5.10 ± 3.14	7.88 ± 2.23	0.060
**Lesion size (cm)**	1.23 ± 0.39	1.12 ± 0.66	0.675
**Diagnostic yield (%)**	**66.7**	**84.2**	**0.562**

## Data Availability

The datasets generated during and/or analyzed during the current study are not publicly available due to security and ongoing research. The data underlying this article will be shared upon reasonable request to the corresponding author.
